# Supplementary figures and images for: Closed-loop optogenetic activation of peripheral or central neurons modulates feeding in freely moving Drosophila
Source: eLife. 2019 Jul 19;8:e45636. doi: 10.7554/eLife.45636 (PMC6668987; doi:10.7554/eLife.45636)

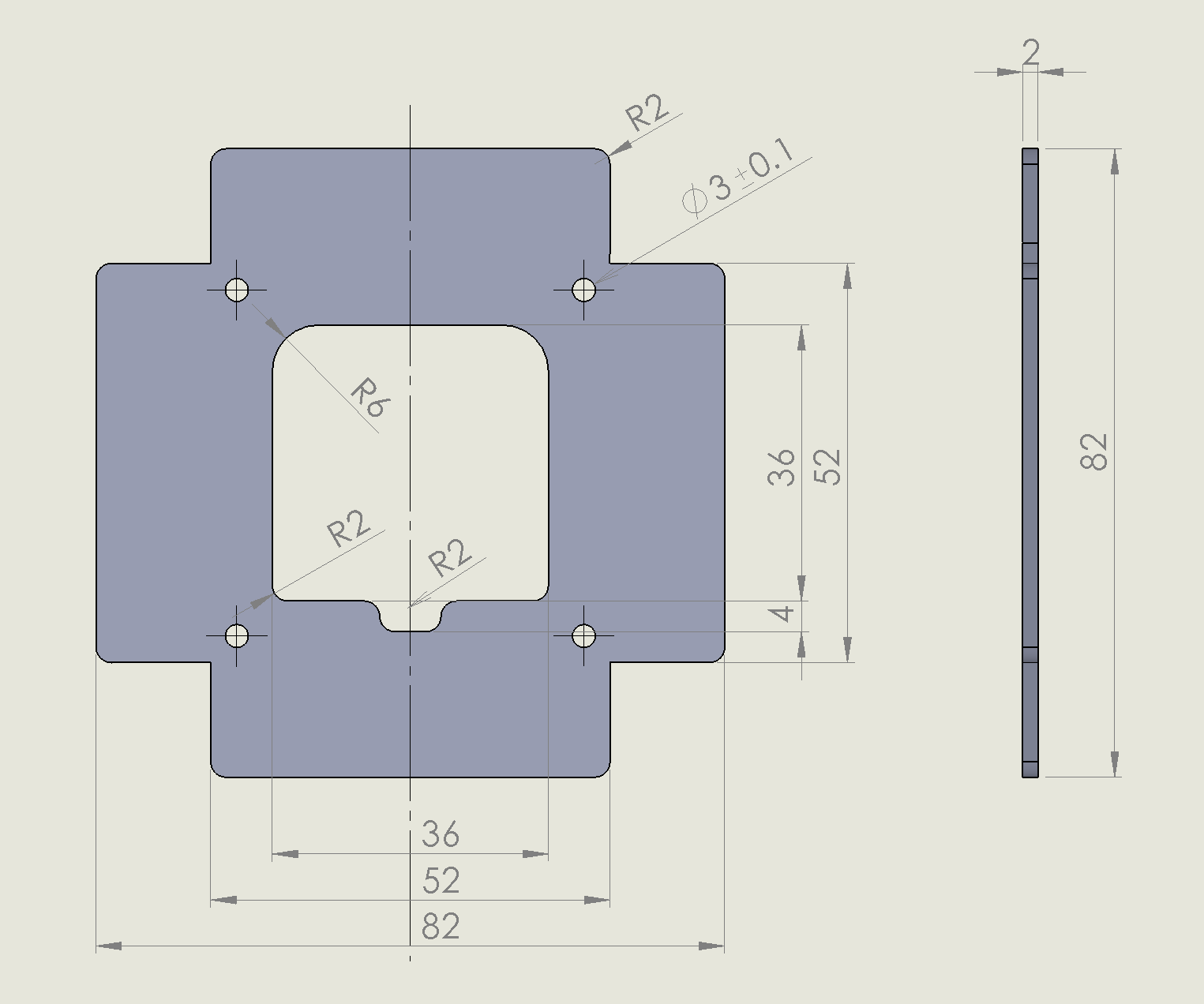

Supplement: Supplementary file 1. [file elife-45636-supp1.zip › STROBE Assembly Package/Housing Fabrication/Housing Mechanical Dimensions.png]

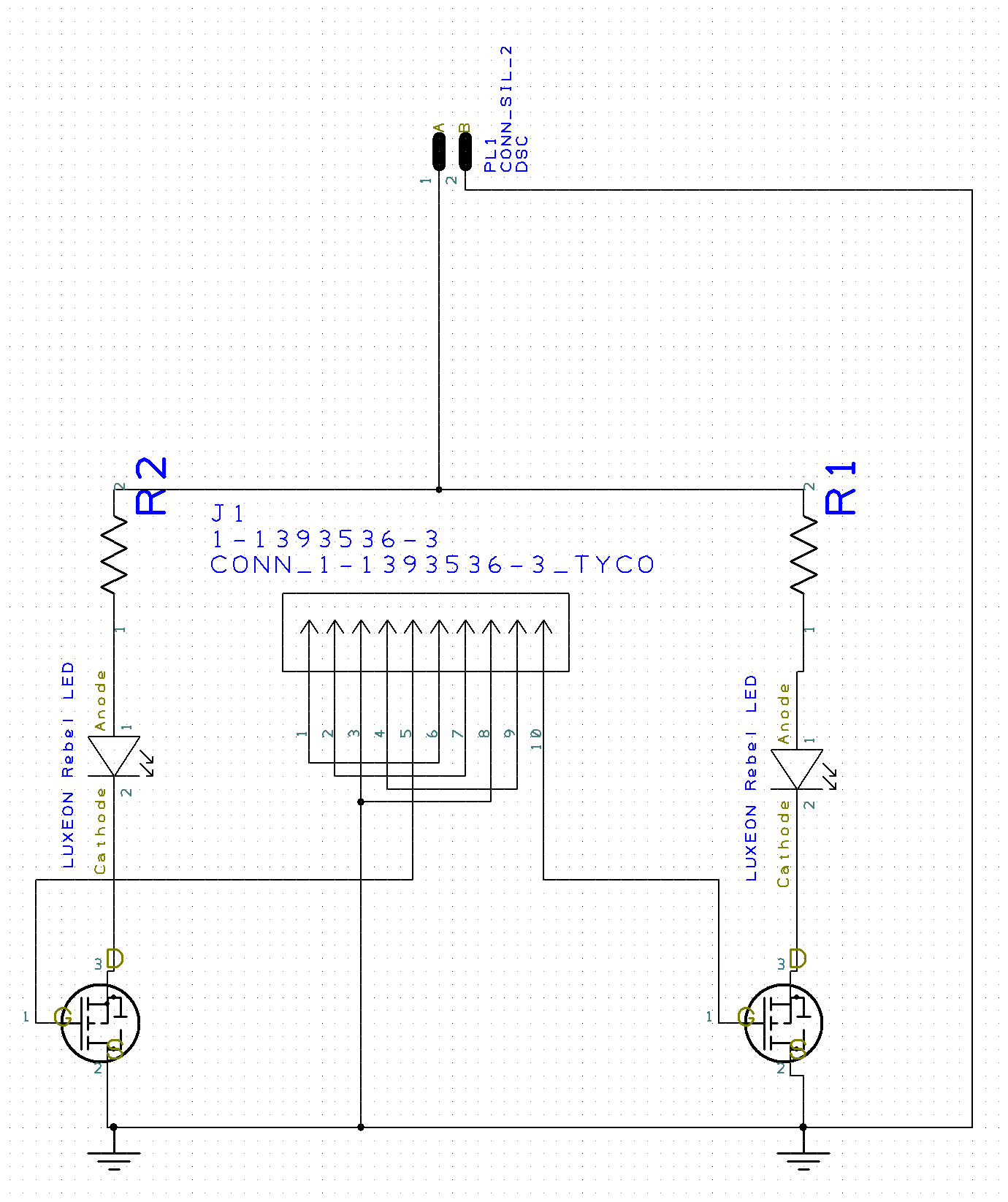

Supplement: Supplementary file 1. [file elife-45636-supp1.zip › STROBE Assembly Package/Lighting Circuit Schematic.png]

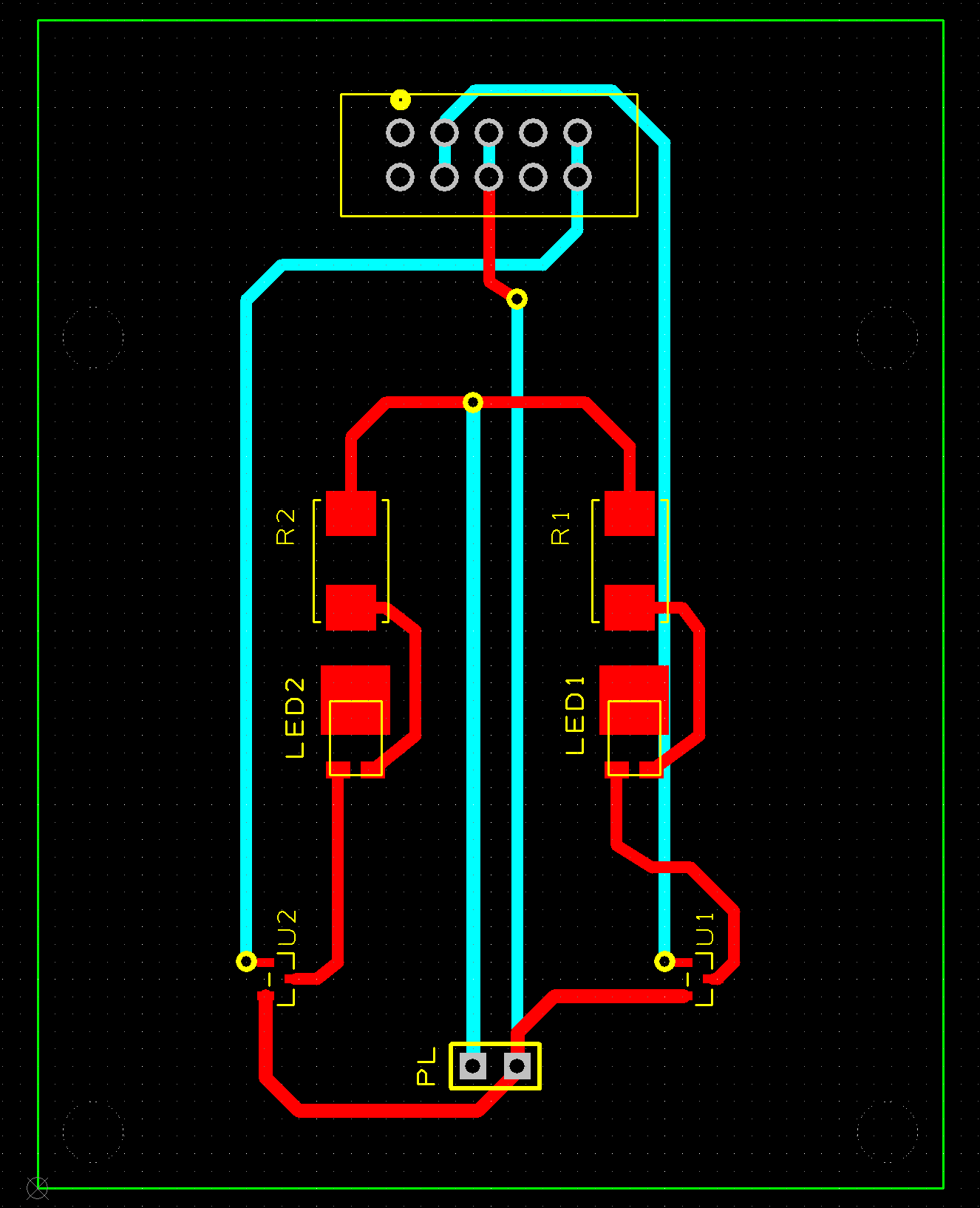

Supplement: Supplementary file 1. [file elife-45636-supp1.zip › STROBE Assembly Package/Lighting PCB Layout.png]

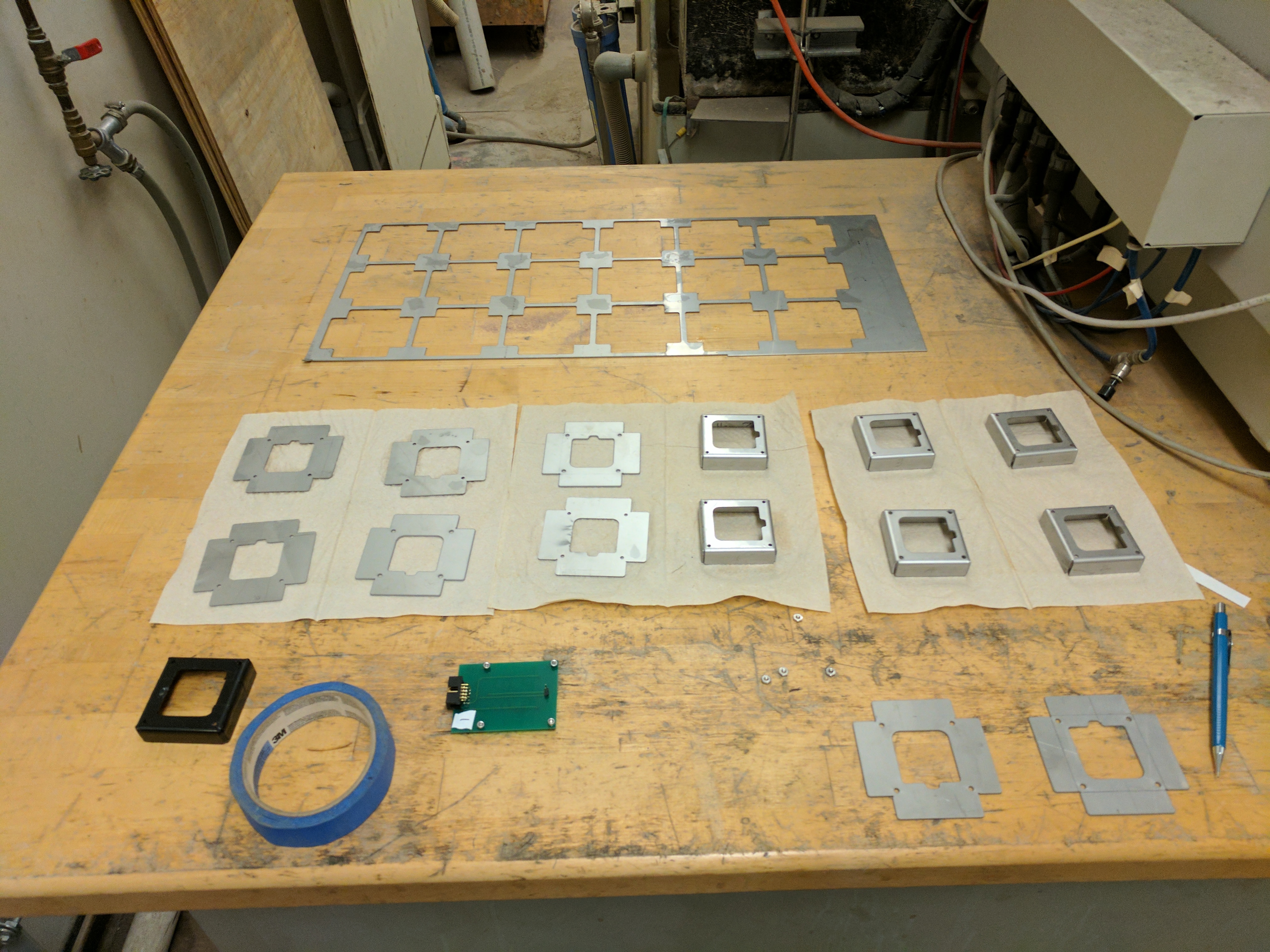

Supplement: Supplementary file 1. [file elife-45636-supp1.zip › STROBE Assembly Package/PCB Housing Fabrication Steps.jpg]

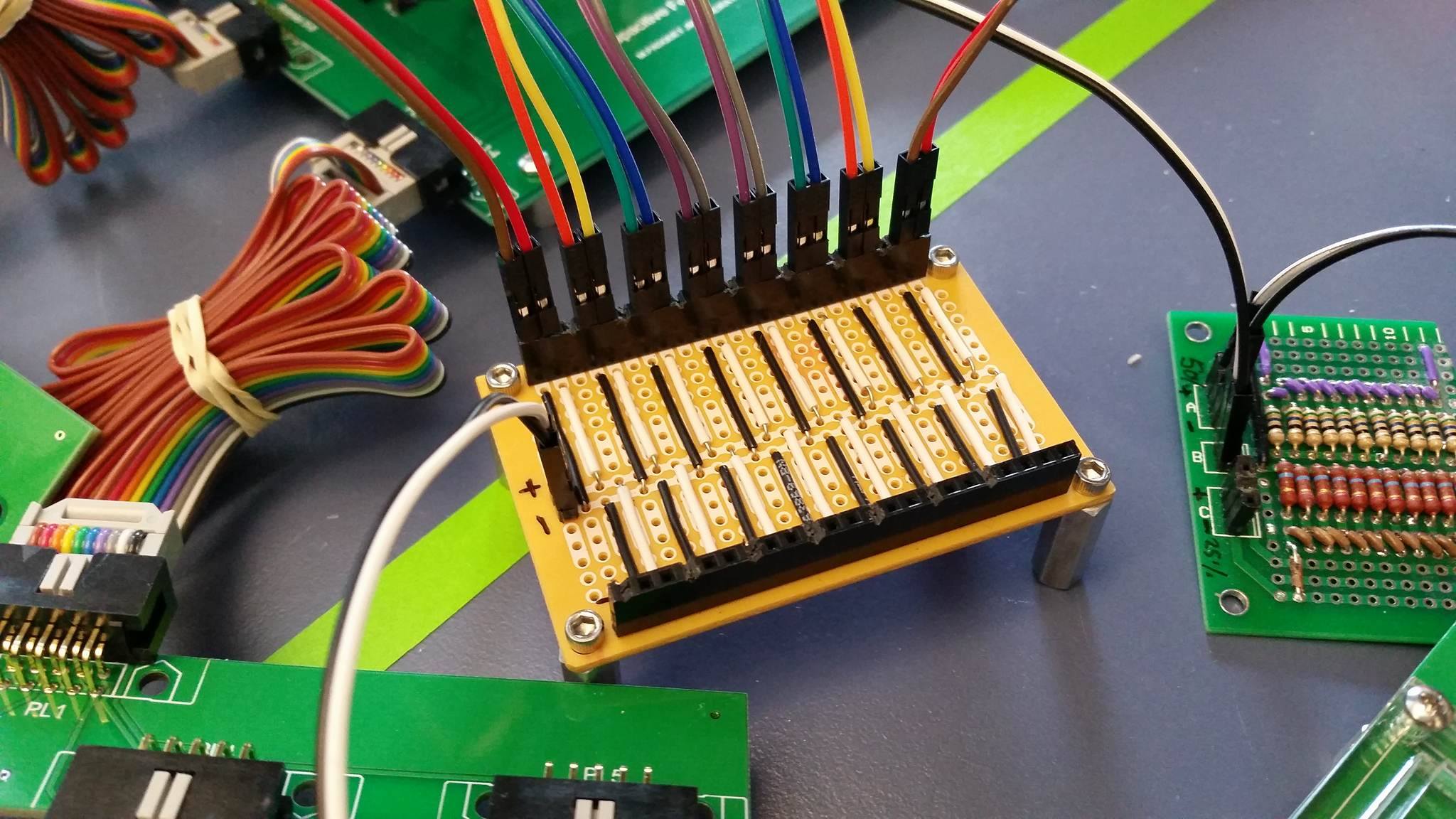

Supplement: Supplementary file 1. [file elife-45636-supp1.zip › STROBE Assembly Package/Power Splitter Board.jpg]

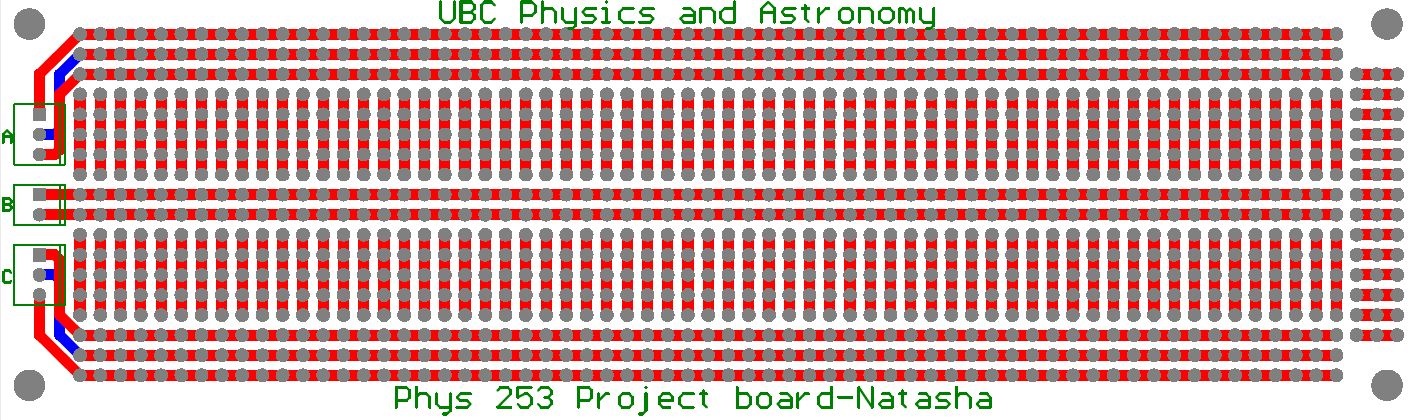

Supplement: Supplementary file 1. [file elife-45636-supp1.zip › STROBE Assembly Package/Project Board Natasha.jpg]

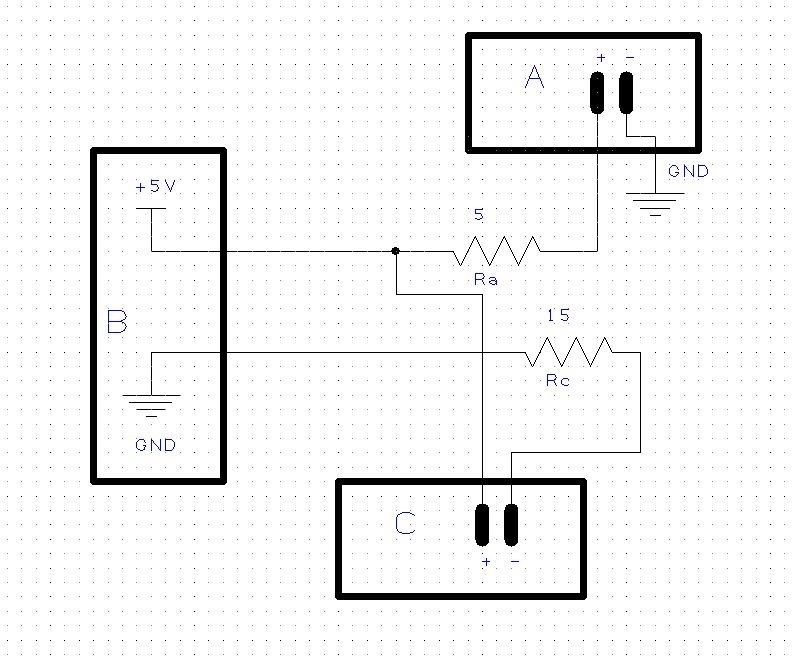

Supplement: Supplementary file 1. [file elife-45636-supp1.zip › STROBE Assembly Package/Resistor Board Schematic.jpg]

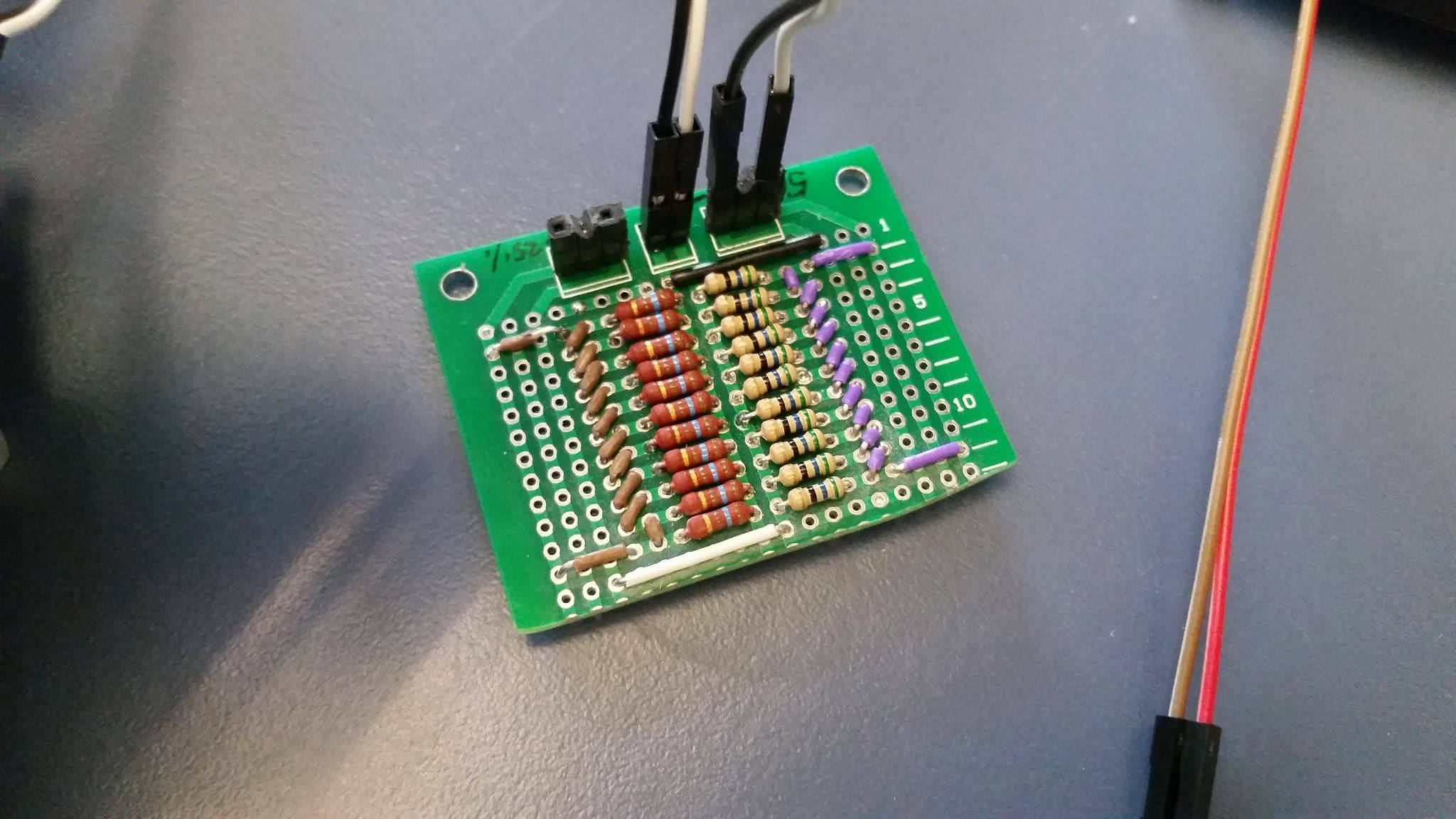

Supplement: Supplementary file 1. [file elife-45636-supp1.zip › STROBE Assembly Package/Resistor Board.jpg]

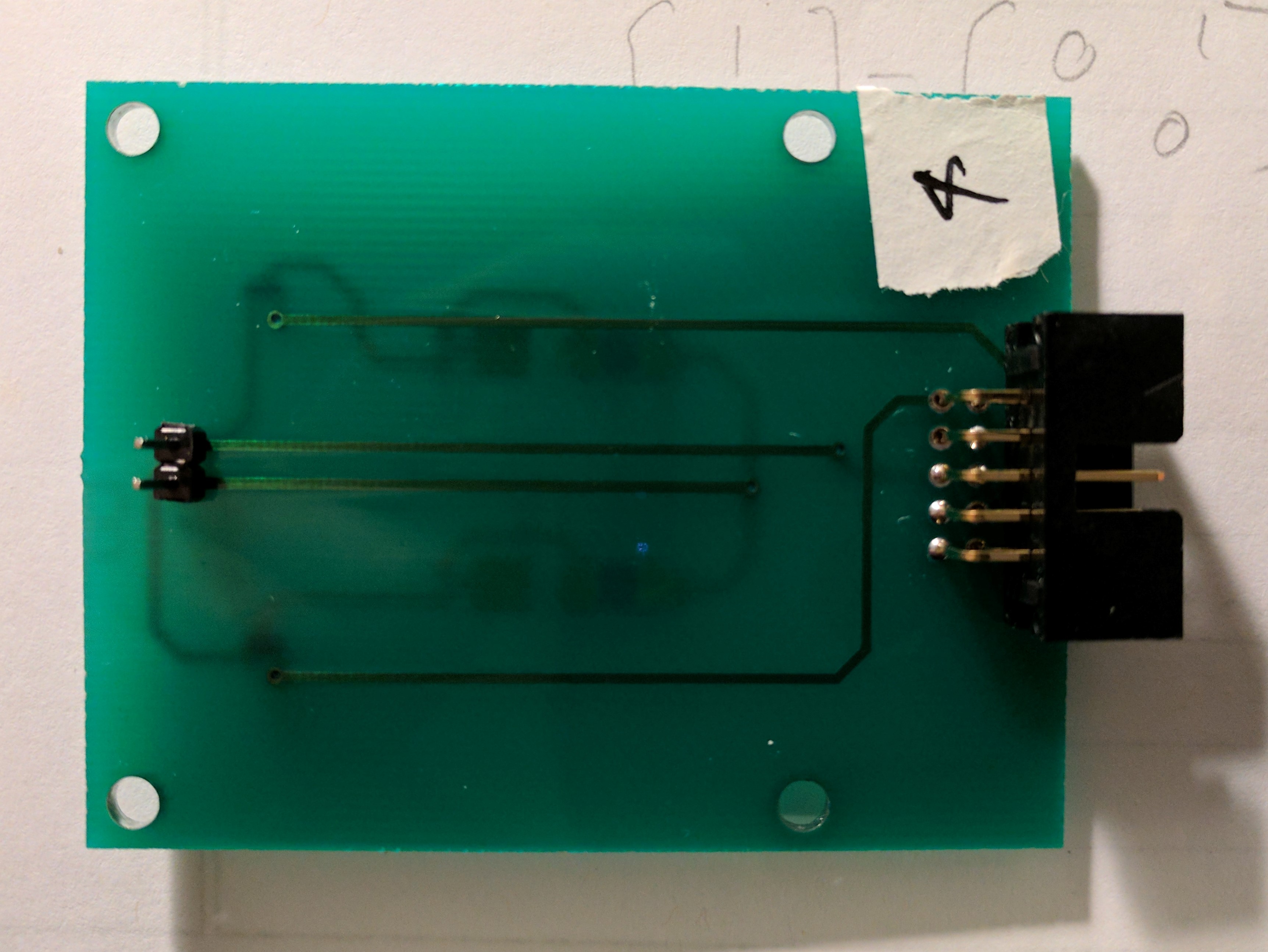

Supplement: Supplementary file 1. [file elife-45636-supp1.zip › STROBE Assembly Package/Soldered PCB Back.jpg]

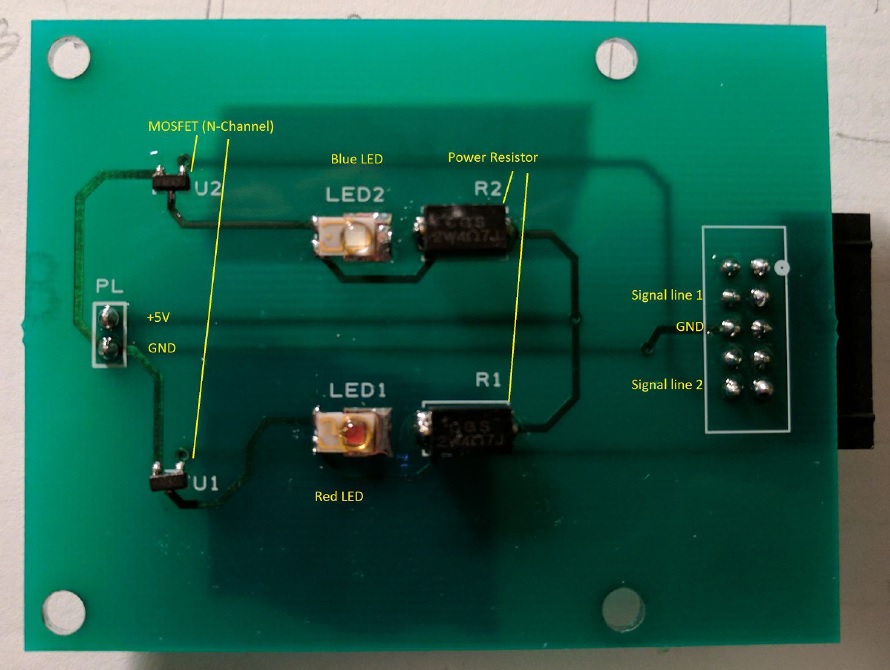

Supplement: Supplementary file 1. [file elife-45636-supp1.zip › STROBE Assembly Package/Soldered PCB Front.jpg]

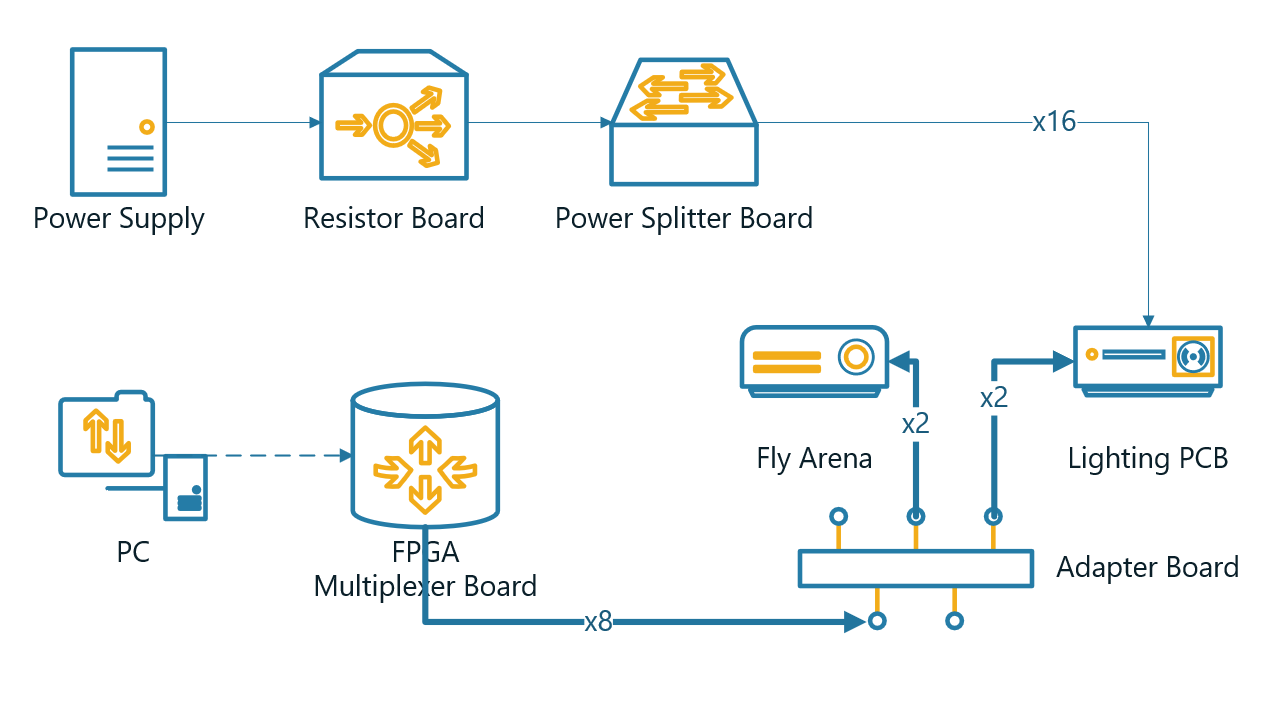

Supplement: Supplementary file 1. [file elife-45636-supp1.zip › STROBE Assembly Package/STROBE Assembly Package/STROBE System Connections Diagram.png]
